# Status Epilepticus due to Severe HHV-6 Encephalitis in an Allogeneic Stem Cell Transplant Recipient

**DOI:** 10.4084/MJHID.2014.008

**Published:** 2014-01-01

**Authors:** Poorvi Chordia, Pranatharthi Chandrasekar

**Affiliations:** Division of Infectious Diseases, Department of Internal Medicine. Wayne State University, Detroit, MI, USA.

## Abstract

Reactivation of human herpes virus-6 (HHV-6) after stem cell transplantation occurs frequently. It is associated with clinical manifestations varying from nonspecific symptoms such as fevers or rash, to severe life threatening complications including post-transplantation limbic encephalitis. We report a case of severe HHV-6 encephalitis with viremia in an allogeneic peripheral stem cell transplant recipient who presented with status epilepticus unresponsive to antiepileptic therapy. With intravenous ganciclovir and supportive care, the patient’s condition improved. Awareness of HHV-6 infection in stem cell transplant recipients may help with early diagnosis and improved outcome.

## Introduction

HHV-6 reactivation has been increasingly recognized in stem cell transplant recipients.^1^ Primary infection with HHV-6 usually occurs during infancy with reactivation in 40–60% patients after stem cell transplantation.^1^ Clinical manifestations of HHV-6 reactivation in stem cell transplantation recipients are variable, including fever, rash, diarrhea, pneumonia, hepatitis, encephalitis, delayed platelet recovery, graft versus host disease (GVHD), and increased mortality.^2^ Median onset of symptoms is about three weeks after stem cell transplant, and often coincides with engraftment.^3^ Our case involves a patient with myelodysplastic syndrome who underwent an allogeneic stem cell transplantation following which he developed severe HHV-6 encephalitis causing status epilepticus.

## Case Report

A 68-year-old man with myelodysplastic syndrome (MDS) secondary to treatment with capecitabine-oxaliplatin for colon cancer, underwent a matched, unrelated donor peripheral stem cell transplantation. Conditioning regimen comprised of fludarabine, busulfan and total body irradiation. For GVHD prophylaxis, the patient received tacrolimus, mycophenolate and thymoglobulin. Stem cell donor was Cytomegalovirus (CMV) seronegative, while the patient was CMV seronegative and Herpes Simplex Virus (HSV) seropositive.

On day +4 after transplantation, cefepime was started for fever during neutropenia. On day +8, the patient developed a generalized non-blanching erythematous maculopapular rash and loose stools. Engraftment syndrome was suspected, and a short course of intravenous steroid therapy was started. The patient engrafted on day +14.

On day +18, the patient developed confusion and anterograde amnesia. Over the next three days, he developed increasing lethargy and multiple generalized tonic-clonic seizures. A CT scan of the head showed no abnormalities. On day +21, the patient developed status epilepticus, unresponsive to antiepileptic medications. Due to declining mental status and to protect his airway, he was endotracheally intubated. For treatment of seizures, therapy with levetiracetam was started.

**Figure 1:**
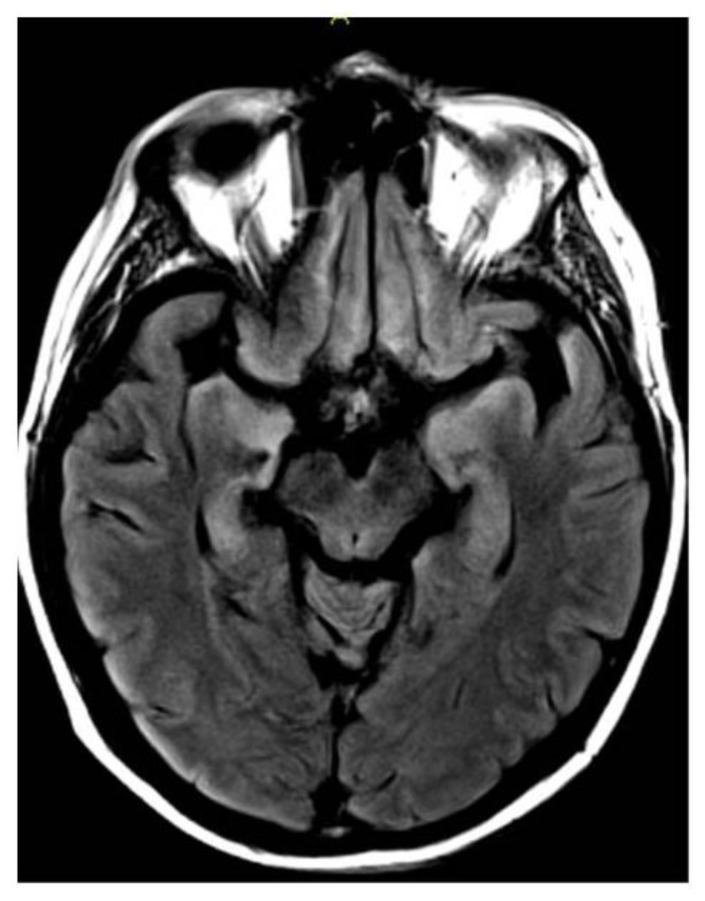
MRI Brain: Increased symmetric T2/FLAIR signal in medial temporal, hippocampi, medial thalamus, and insular regions

Video EEG demonstrated bilateral central-frontal slow epileptiform waves with clinical manifestations of right arm jerking that spread to bilateral lower extremities. Cerebrospinal fluid (CSF) analysis showed normal levels of protein and glucose, and no pleocytosis. CSF bacterial cultures as well as CSF polymerase chain reaction (PCR) for Herpes Simplex Virus, West Nile Virus, Epstein Barr Virus and Varicella Zoster Virus were negative. Serum tacrolimus level was within normal limits. MRI brain showed increased symmetric T2/FLAIR signal in medial temporal lobes, hippocampi, medial thalamus, and insular regions (Figure 1). Plasma and CSF HHV-6 DNA PCR were 57,000 copies/ml and 81,796 copies/ml, respectively. Based on clinical and characteristic radiographic findings along with HHV-6 PCR, the diagnosis of HHV-6 encephalitis was made.

The patient was treated in the intensive care unit with ventilator support, anticonvulsants, intravenous ganciclovir (5mg/kg twice daily). Gradually, his seizures subsided and he was subsequently extubated. He continued to have progressive improvement, although mild anterograde amnesia was observed even after ten days of antiviral treatment. Repeat plasma HHV-6 DNA PCR at two weeks was negative, and intravenous ganciclovir was discontinued after a total treatment of 3 weeks.

## Discussion

HHV-6 encephalitis is a sporadic complication of HHV-6 reactivation in stem cell transplant recipients. Our patient had early features of HHV-6 reactivation as evidenced by fevers and rash on day +8 after transplantation. His condition progressed to encephalitis with symptoms of confusion and anterograde amnesia on day +18, and status epilepticus on day +21. The severity of our patient’s neurologic manifestations could be explained by the long clinical evolution. In a study by Fujimaki et al., 9 of 11 patients with HHV-6 encephalitis had herald symptoms of fevers and rash prior to the onset of neurologic signs and symptoms.^4^ Prodromal symptoms of fevers and rash in this setting should alert the physician of possible HHV-6 reactivation.

Similar to our patient, analysis of CSF in HHV-6 encephalitis is usually unremarkable. Lack of pleocytosis and normal levels of protein and glucose are common.^5^ The presence of a positive CSF HHV-6 PCR, with suggestive clinical findings clinches the diagnosis. The patient also had characteristic MRI findings of limbic encephalitis which is typical for HHV-6 encephalitis.^6^

As with the cytomegalovirus PCR, the international standardization of HHV-6 PCR quantification is urgently needed. There are no data correlating quantification of PCR with severity of symptoms. Our patient had a “high” viral load in the serum and CSF, perhaps suggesting severe illness. In a study by Ogata et al., it was found that plasma HHV-6 DNA ≥10,000 copies/ml correlated with the development of HHV-6 encephalopathy and offered 100% sensitivity and 64.6% specificity.^7^

Although it remains uncertain if HHV-6 reactivation after stem cell transplantation increases mortality, the prognosis associated with HHV-6 encephalitis remains poor. In one study, 80% patients with HHV-6 encephalitis post-transplantation were disabled because of persistent neuropsychological problems that included retrograde amnesia and seizures.^8^

Treatment for HHV-6 encephalitis includes intravenous ganciclovir, foscarnet or cidofovir. Acyclovir is not active against HHV-6. Due to poor outcomes secondary to HHV-6 reactivation, the usefulness of preemptive^9^ or prophylactic therapy^10^ needs to be determined. Currently, the European Conference on Infections in Leukemia does not recommend routine prophylaxis with potentially toxic ganciclovir or foscarnet against HHV-6 reactivation in patients who have undergone stem cell transplantation.^11^

HHV-6 reactivation after stem cell transplantation is not uncommon, and can often lead to potentially lethal complications such as limbic encephalitis. More data are needed to determine the benefits of routine surveillance and pre-emptive therapy, especially in those patients at high risk to reactive HHV-6, as seen with cord blood transplant recipients.
